# Differential Kat3 Usage Orchestrates the Integration of Cellular Metabolism with Differentiation

**DOI:** 10.3390/cancers13235884

**Published:** 2021-11-23

**Authors:** Xiaohui Hu, Masaya Ono, Nyam-Osor Chimge, Keisuke Chosa, Cu Nguyen, Elizabeth Melendez, Chih-Hong Lou, Punnajit Lim, John Termini, Keane K. Y. Lai, Patrick T. Fueger, Jia-Ling Teo, Yusuke Higuchi, Michael Kahn

**Affiliations:** 1 Department of Pathology, School of Basic Medical Sciences, Anhui Medical University, Hefei 230032, China; huxiaohui@ahmu.edu.cn; 2 Department of Molecular Medicine, Beckman Research Institute of City of Hope, Duarte, CA 91010, USA; nchimge@coh.org (N.-O.C.); kchosa@coh.org (K.C.); cunguyen@coh.org (C.N.); emelendez@coh.org (E.M.); plim@coh.org (P.L.); jtermini@coh.org (J.T.); klai@coh.org (K.K.Y.L.); jteo@coh.org (J.-L.T.); yhiguchi@coh.org (Y.H.); 3 Department of Clinical Proteomics, National Cancer Center Research Institute, Tokyo 104-0045, Japan; masono@ncc.go.jp; 4 Gene Editing and Viral Vector Core, Beckman Research Institute of City of Hope, Duarte, CA 91010, USA; clou@coh.org; 5 City of Hope Comprehensive Cancer Center, Duarte, CA 91010, USA; pfueger@coh.org; 6 Department of Molecular and Cellular Endocrinology, Beckman Research Institute of City of Hope, Duarte, CA 91010, USA

**Keywords:** CBP, p300, metabolism, glycolysis, OXPHOS

## Abstract

**Simple Summary:**

The coupling of metabolism with cellular status is critically important and highly evolutionarily conserved. However, how cells coordinate metabolism with transcription as they change their status is not clear. Utilizing multiomic and functional studies, we now demonstrate the dichotomous roles of the Kat3 coactivators CBP and p300 and, in particular, their extreme N-termini, in coordinating cellular metabolism with cell differentiation. Using multiple in vitro and in vivo systems, our study sheds new light on metabolic regulation in homeostasis and disease, including cancer.

**Abstract:**

The integration of cellular status with metabolism is critically important and the coupling of energy production and cellular function is highly evolutionarily conserved. This has been demonstrated in stem cell biology, organismal, cellular and tissue differentiation and in immune cell biology. However, a molecular mechanism delineating how cells coordinate and couple metabolism with transcription as they navigate quiescence, growth, proliferation, differentiation and migration remains in its infancy. The extreme N-termini of the Kat3 coactivator family members, CBP and p300, by far the least homologous regions with only 66% identity, interact with members of the nuclear receptor family, interferon activated Stat1 and transcriptionally competent β-catenin, a critical component of the Wnt signaling pathway. We now wish to report based on multiomic and functional investigations, utilizing p300 knockdown, N-terminal p300 edited and p300 S89A edited cell lines and p300 S89A knockin mice, that the N-termini of the Kat3 coactivators provide a highly evolutionarily conserved hub to integrate multiple signaling cascades to coordinate cellular metabolism with the regulation of cellular status and function.

## 1. Introduction

The integration of a cell’s state with its metabolism is critically important and the coupling of energy production and cellular function is highly evolutionarily conserved. This has been demonstrated in stem cell biology, organismal, cellular and tissue differentiation and, perhaps most extensively, in immune cell biology [1,2,3]. However, a molecular understanding of how cells coordinate and couple metabolism with transcription as they navigate quiescence, growth, proliferation, differentiation and migration remains in its infancy. Metabolism is driven by the expression of specific enzymatic products and gene expression and protein translation requires the continual production of certain metabolites and ATP. 

The Wnt signaling cascade plays a significant role in the maintenance, proliferation and differentiation of stem/progenitor cells [4,5,6] as well as in regulating multiple metabolic parameters, including glucose metabolism, de novo lipogenesis and mitochondrial physiology [7,8]. Similarly, the nuclear receptor family is an essential component of stem/progenitor cell biology, regulating both the maintenance of stemness, differentiation and lineage commitment [9]. Nuclear receptor family members critically control key nutrient pathways, including fatty acid oxidation, gluconeogenesis in the fasted state and lipogenesis and glycolysis [10]. For example, a very recent report outlined the critical role of several nuclear receptors, including PPAR-α and ESRRA, in proximal tubule kidney cells, via coordination of metabolism and differentiation and their dysfunction in kidney fibrosis [11]. Numerous studies have documented a significant profibrotic role for aldosterone, via the mineralocorticoid receptor, in the progression of chronic kidney disease [12,13]. Wnt signaling has also been associated with kidney cell differentiation [14] as well as with aberrant differentiation and renal fibrosis [15]. Similarly, interferon via activation of the Janus kinase (JAK)-signal transducer and activator of transcription 1 (STAT1) pathway induces the expression of genes that have key immune effector functions and key metabolic roles to regulate immunometabolism and immune cell polarization [16]. Interferon-γ also possesses antifibrotic effects via differentiation [17]. However, interferon-γ signaling contributes to renal fibrosis and chronic kidney disease progression [18]. Clearly, multiple signaling cascades converge on the regulation of metabolism and differentiation to maintain homeostasis or to resolve inflammatory processes after injury, and aberrant coordination leads to diseases, including fibrosis, cancer and neurodegenerative diseases. 

How do these pathways intersect and how are these dichotomous outcomes regulated? The Kat3 coactivators, CBP and p300, diverged via a gene duplication just prior to the vertebrate radiation over 450 million years ago [19]. They encode extremely large proteins with molecular weights of approximately 300 kDa, over 33 and 31 exons, respectively. They retain an extremely high degree of identity, up to 93%, particularly over a large central core that includes the CH1, KIX, Bromodomain and CH2 and CH3 regions (Figure S1A) [20,21]. They interact with hundreds of proteins in their roles as master orchestrators of transcription. Perhaps not surprisingly, they have long been considered largely redundant due to their high degree of protein sequence identity and even higher similarity. However, it is now clear that CBP and p300 play unique and definitive roles both in vitro and in vivo [4,22,23,24,25]. Intriguingly, the extreme N-termini of the Kat3 coactivator family members, CBP and p300, by far the least homologous regions with only 66% identity, interact with members of the nuclear receptor family via a conserved LXXLL sequence, interferon activated Stat1 and transcriptionally competent β-catenin, a critical component of the Wnt signaling pathway [4,26], thereby providing a hub to integrate multiple signaling cascades to coordinate cellular metabolism with the regulation of cellular status. 

We have previously proposed an evolutionary rationale for the rapid divergence of the two Kat3 coactivators from one another within their N-terminal 111 amino acid regions, yet within each orthologous group retaining 98% identity at the amino acid level within these regions from mouse to human [4]. We previously proposed that this provided a high-fidelity mechanism for the long-term homeostatic maintenance and repair that long-lived complex vertebrates require. N-terminal CBP transcriptional complexes (i.e., at super enhancers, enhancers and promoters) [24,27,28] help to maintain an anaerobic quiescent state that is critical to maintain the integrity of the genetic material in the quiescent somatic stem cell (SSC) pool via asymmetric differentiation, whereas a switch to p300 N-terminal transcriptional complexes, orchestrates the gene expression cassettes and metabolic requirements of differentiated daughter cells [4,29]. We previously demonstrated, utilizing CRISPR/Cas9 editing [30], that one significant difference in the highly evolutionarily conserved N-termini of CBP and p300, a 27 bp/9aa deletion in CBP, between the β-catenin-binding region (DELI-sequence) and the nuclear receptor binding sequence (LXXLL), provided a mechanism for nuclear receptors, via steric inhibition, to cleanly antagonize CBP/β-catenin signaling, thereby either maintaining SSC quiescence or initiating asymmetric divisions. Whereas β-catenin, when bound to p300, is not sterically constrained from binding nuclear receptor family members, thus allowing for synergy to affect a feed-forward mechanism to drive differentiation and lineage commitment [30]. The N-terminal domains of CBP and p300, within the first 111 amino acids, additionally contain 15 and 19 serine and threonine residues, respectively, providing a rich environment for post-translational modification via phosphorylation/dephosphorylation signaling cascades [4]. 

To date, our attention has been primarily focused on serine 89 (S89) in p300. P300 S89 is a substrate for a number of kinases, including PKC, AMPK and SIK2, associated with an array of biological effects, including activation and inhibition of transcription, inhibition of histone acetyltransferase function, metabolic regulation of insulin/glucagon signaling and carbohydrate-responsive element-binding protein (ChREBP) control of glycolysis and lipogenesis [31,32,33,34,35,36,37] and the differentiation of ES cells and adult progenitor cells [35,38]. More recently, we have generated p300 S89A knockin mice. Global genomic and proteomic analyses revealed major pathway differences including lipid metabolism, oxidative stress response, mitochondrial function and oxidative phosphorylation, with a diverse array of effects on fundamental processes including epithelial differentiation, metabolism, immune response and microbiome colonization, all brought about by this single amino acid modification [39]. Thus, the critical role of this signaling nexus and the rationale for its evolutionary conservation is highlighted. We now wish to report based on additional multiomic investigations, utilizing p300 knockdown (KD), N-terminal p300 edited and p300 S89A edited cell lines and p300 S89A knockin mice, that differential N-terminal Kat3 coactivator usage is a highly evolutionarily conserved mechanism to couple metabolism and energy production to cellular state and function.

## 2. Materials and Methods

### 2.1. Cell Culture

MDA-MB-231 and C2C12 were cultured in Dulbecco’s Modified Eagle Medium (DMEM) with 10% fetal bovine serum (FBS) and 1% Penicillin-Streptomycin (PS) added. To induce C2C12 differentiation, cells were cultured in DMEM with 2% horse serum plus 1% PS for 2 days. P19 cells were cultured in MEM alpha medium containing 7.5% bovine calf serum and 2.5% FBS, with 1% PS. To induce P19 differentiation, cells were treated with DMEM + 10% FBS + 1% PS plus 1 µM retinoic acid (RA) for 5 days. To test MDA-MB-231 cell proliferation in different media conditions, cells were cultured in control DMEM medium described above, 2-DG medium (control DMEM medium plus 5 mM 2-DG) or galactose medium prepared from no glucose DMEM medium (Sigma, D5030-10 × 1L, Saint Louis, MO, USA,) supplemented with 4 mM L-glutamine, 10 mM galactose, 1 mM sodium pyruvate, 1 mM HEPES, 1% PS and 10% FBS.

### 2.2. Proteomic Sample Preparation and Mass Spec Analysis 

To prepare the samples, cells were treated, harvested and washed with PBS. Then, 100% methanol was added to the cells and fixed at room temperature for ten minutes. After fixation, the methanol was dried using a speed-vac/lyophilizer setup for 30 min at 40 °C. The dried cell samples were then resuspended in 200 µL of 2% SDC, 80 µL of 5M Urea, 20 µL of NH_4_HCO_3_ and 95 µL of water with 2 µg of trypsin (Promega, V5280, Madison, WI, USA) and subjected to trypsin digestion in a 37 °C on rotating incubator for 20 h. A total of 80 µL of 5% formic acid was added to the digested sample and vortexed to precipitate undigested proteins. The sample was then spun at 15,000× *g* for 10 min to pellet solid material. The solution was transferred to a clean 1.5 mL Eppendorf tube and 480 µL of ethyl acetate was added. After mixing, the sample was spun again at 15,000× *g* for 10 min and the top ethyl acetate layer was removed by aspiration. The peptide sample was then dried up overnight on a speed-vac/lyophilizer setup at 37 °C and then suspended in 50 µL of 0.1% formic acid, with the peptide concentration determined using the GE NanoVue Plus spectrophotometer. A total of 10 µg of peptide was passed through a C18 spin column (Thermo Fisher, 84850, Waltham, MA, USA) and washed twice with 20 µL of 2% acetonitrile, 0.1% formic acid each time. Bound peptide was eluted from the C18 column twice with 20µL of 80% acetonitrile, 0.1% formic acid each time. The sample was evaporated overnight and then resuspended in 0.1% formic acid to take a final concentration read on the GE NanoVue Plus spectrophotometer. Finally, the samples were dried overnight on the speed-vac/lyophilizer prior to being sent out, dried at room temperature for mass spec analysis. Tissues were extracted from mice and flash frozen with liquid nitrogen. Frozen tissues were crushed with a mortar and pestle while frozen and then stored at −80 °C until ready for trypsin digestion. Then, 100% methanol was added to the thawed, crushed tissue and fixed at room temperature for ten minutes. The fixed tissue was then subjected to the same protocol for trypsin digestion as the cell protocol above. The mass spec analysis was performed as previously described [30].

### 2.3. Western Blot Analysis

Whole cell lysates were collected with RIPA buffer. To test the expression of CBP and p300, nuclear lysates were prepared with a NE-PER™ Nuclear and Cytoplasmic Extraction kit (Thermo Fisher, 78835). After lysate clarification, the protein concentration of the supernatant was measured by Bio-Rad protein assay (Bio-Rad, #500-0006, Hercules, CA, USA). Protein extracts were mixed with SDS loading buffer and boiled for 5 min before loading. Subsequently, 20 ug of proteins per lane was loaded into pre-cast 4–20% gradient gels (Expedeon, BCG42012, Cambridgeshire, UK), accompanied with tricolor pre-stained protein markers. After gel electrophoresis, proteins were transferred onto methanol activated PVDF membranes overnight at 25 V at 4 °C. The membranes were blocked with 5% skim milk at RT for 1 h, followed by primary antibody incubation overnight at 4 °C. The primary antibodies used in this study were: ENOA (CST, 8866S, 1:1000, Danvers, MA, USA); ALDOA (CST, 3188S, 1:1000); ALDOC (Santa Cruz, sc-271593, 1:200, Dallas, TX, USA); G3P (Santa Cruz, sc32233, 1:1000); H3 (Santa Cruz, sc10809, 1:2000); CBP (Aviva, ARP43609-P050, 1:1000, San Diego, CA, USA); EP300 (Aviva, OAAF01891, 1:1000); TOM20 (Santa Cruz, sc17764, 1:100). On the next day, membranes were washed in 0.1% TBST buffer, 3 times at 5 min intervals, followed by the corresponding secondary antibody incubation for 1 h at RT. Membranes were washed 4 times at 5 min intervals and then subjected to Amersham ECL Prime Western Blotting Detection Reagent (RPN2232). The band signal intensities were detected using a ChemiDoc Imaging System from Bio-Rad. 

### 2.4. Quantitative RT-PCR Analysis

Total RNA was extracted with TRIzol reagent (Thermo Fisher) following the manufacturer’s instructions. A total of 2 µg of total RNA was subjected to reverse transcription to cDNA using a qScript cDNA Synthesis Kit (Quantabio, 95047-100, Beverly, MA, USA). To test a specific gene’s expression, 0.5 µL of cDNA was added to the quantitative PCR (qPCR) reaction, together with specific primers and 2× SYBR Green RT-PCR Master Mix. A qPCR was performed on a Bio-Rad CFX connect optics module. Data were analyzed by the 2^^-ΔΔCt^ value. All the primers sequences are listed in Table S1.

### 2.5. Seahorse Analysis of Glycolysis and Mitochondrial Metabolism 

Cells were seeded at an optimized density of 20,000 cells per well and incubated in their corresponding growth medium for 24 h before assays were performed. For C2C12 differentiation, cells were incubated under four conditions: growth medium, differentiation medium (DM), DM with 5 µM ICG-001 or DM with 2 µM YH250. Metabolic profiles were generated on the Seahorse XF96 Extracellular Flux Analyzer (Agilent, Santa Clara, CA, USA) according to manufacturer’s instructions. ATP rate assays were performed with subsequent injections of 1.5 µM oligomycin in port A and 0.5 µM rotenone and antimycin A in port B. Glycolytic rate assays were performed with subsequent injections of 0.5 µM rotenone and antimycin A in port A and 50 mM 2-DG in port B. Mitochondrial stress tests were performed with subsequent injections of 1 µM oligomycin in port A, optimized FCCP (1 µM for MDA-MB-231, 4 µM for P19 WT + RA and 2 µM for P19 EDITED + RA cells) in port B and 0.5 µM rotenone and antimycin A in port C. Except for the metabolism inhibitors, 10 mM Hoechst was added to the last injection for all Seahorse assays, to perform cell counting by fluorescence scanning on the Cytation plate reader (BioTek, Winooski, VT, USA) for cell number normalization. The final report was generated with the corresponding Seahorse wave programs.

### 2.6. Immunofluorescence

To visualize mitochondrial morphology, 1 × 10^5^ MDA-MB-231 cells were plated in 12 well plates with coverslips inside. When the cell density reached 70–80% confluency, cells were fixed with warmed 4% PFA and permeabilized with 0.2% tritonX-100. The coverslips were taken out and placed in the incubation chamber with wet papers. A total of 1% BSA was used to block the cells for 1 h at RT. Primary antibody TOM20 (Santa Cruz, sc-17764, 1:100) was added and diluted in 0.5% BSA and incubated overnight at 4 °C. On the next day, the cells were washed with PBS three times and incubated with the secondary antibody Alexa Fluor 568 Goat anti-Mouse antibody (Invitrogen, A11031, Carlsbad, CA, USA) at RT for 1 h. The coverslips were mounted with VECTASHIELD mounting medium with DAPI (VECTOR, H-1200). Images were taken on a Zeiss LSM 880 confocal microscope with oil lens.

### 2.7. Transmission Electron Microscopy (TEM) 

MDA-MB-231 control, CBP KD and p300 KD cells were collected, and the cell pellets were fixed with 2% glutaraldehyde in 0.1 M Cacodylate buffer (Na(CH_3_)_2_AsO_2_·3H_2_O), pH 7.2, at 4 °C overnight. Sample preparation was performed at the electron microscopy and atomic force microscope core at City of Hope. Cell images to visualize the mitochondria were collected on a Tecnai 12 Transmission Electron Microscope equipped with a Gatan UltraScan 2K CCD camera at 1100 times magnification.

### 2.8. Mitochondrial Activity and Mass Testing by MitoTracker 

P19 cells were seeded at 5 K/cm^2^ density and grown in glucose or galactose containing medium. After 4 days culture, cells were stained with 100 nM MitoTracker™ Orange (Thermo Fisher, M7510) and 50 nM MitoTracker Green (Thermo Fisher, M7514) for 30 min and analyzed by flow cytometry using Attune^®^ NxT cytometer to detect the signal intensity for MitoTracker Orange and Green, which represent the mitochondrial activity and mass, respectively.

### 2.9. Relative Mitochondrial DNA Content Testing 

Total DNA was extracted using a Puregene Kit A (Qiagen, 158722, Germantown, MD, USA). DNA was diluted into 1 ng/µL, and 0.5 µL of diluted DNA was used for SYBR GREEN qPCR. The qPCR analysis was performed using a primer targeting mtDNA region that amplifies mt-tRNA (forward: CACCCAAGAACAGGGTTTGT, reverse: TGGCCATGGGTATGTTGTTA), and normalized to amplification targeting genomic DNA, ß2M (forward: TGCTGTCTCCATGTTTGATGTATCT, reverse: TCTCTGCTCCCCACCTCTAAGT). Data were analyzed by the ΔΔCt value.

### 2.10. P19 p300 S89A Cell Line Generation by CRISPR/Cas9 Editing System 

CRISPR sgRNAs (crRNA) targeting the p300 exon 2 region that spans the 89th amino acid, serine (S89) locus were designed using GPP sgRNA Designer (https://portals.broadinstitute.org/gpp/public/analysis-tools/sgrna-design, accessed on 27 August 2020). One pair of sgRNAs was selected, CR1 (AGCTCCCCAAACCTCAACAT) and CR2 (AACAGCTGTCAGAACTGCTG), whose cutting efficiency was tested by Surveyor assay [40]. The specific sequence of the donor template p300 S89A was designed with the sgRNA recognition sequence and PAM sequence being mutated to prevent CRIPSR re-cutting, while the amino acid sequences being unchanged except for S89 mutated into alanine, S89A (AGC > GCC). The donor template, pBS-SK-MK-mP300-DT, was co-transfected with both pX458-CR1 and pX458-CR2 vectors into P19 cells. Cells were sorted with GFP/mCherry double positive expression at 48 h after transfection. The sorted cells were cultured in single clone format and the individual clone’s genomic sequence was investigated by PCR sequencing using the genomic DNA extracted (Lucigen, QE09050, Middleton, WI, USA) from the expanded cell clone. The primers set (5′-TGTCTTAAGAGCTTCTGATTTTGGT-3′ and 5′-CATTGCCCATGCCTGCATTT-3′) was used for the sequencing assay. Positive clones were selected according to their sequencing results, which indicated that the 89th serine was mutated into alanine. 

### 2.11. Generation of MDA-MB-231 p300 Knockdown Cells and MDA-MB-231 CBP Knockdown Cells 

The MDA-MB-231 p300 knockdown cells and MDA-MB-231 CBP knockdown cells were generated using the BLOCK-iT™ Lentiviral Pol II miR RNAi Expression System, with miR 807 directed against p300 and miR 516 directed against CBP, respectively, as per protocol of the manufacturer Invitrogen (Carlsbad, CA, USA).

### 2.12. Statistics 

Data for repeats are presented as means and standard deviation. A two-tailed Student’s *t*-test was used to test statistical significance. *p* < 0.05 was set as statistical significance. Proteomics raw data has been quantile normalized and differential expression was analyzed by one-way analysis of variance using Partek Genomics Suite 7.0 (Partek Inc., Saint Louis, MO, USA). Gene Ontology (GO) enrichment analysis implemented enrichment score (negative natural logarithm of the *p*-value derived from the Fisher’s exact test) to detect over-representation of biological process categories. Differentially expressed proteins were further subjected for pathway enrichment using Ingenuity Pathway Analysis [41] package (http://www.ingenuity.com, accessed on June 2020). 

## 3. Results

### 3.1. Differential Roles of the Kat3 Coactivators in Glycolysis

**Figure 1:**
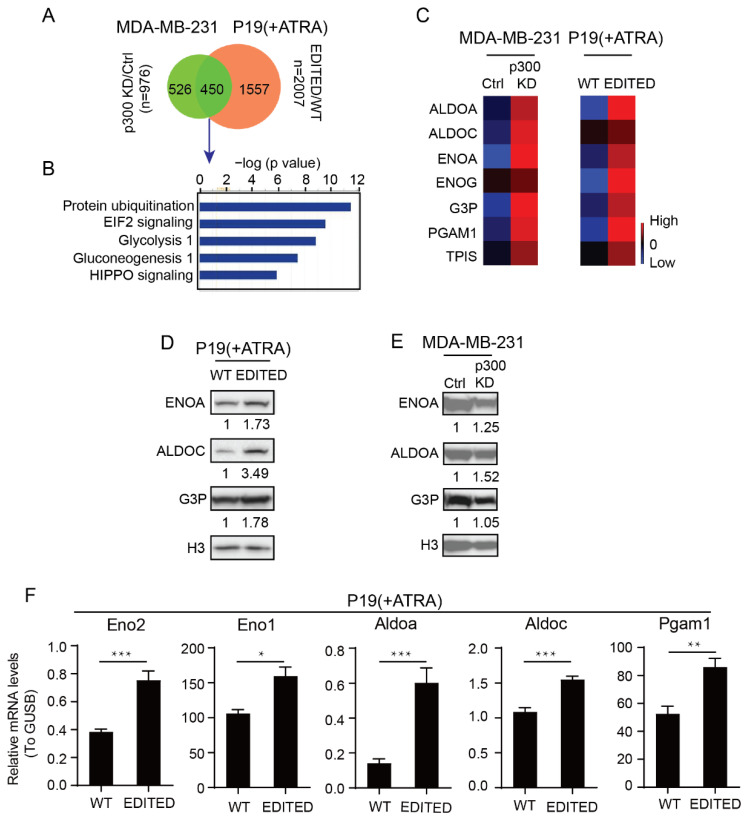
Disrupted p300 function favors glycolytic cell metabolism. (**A**) Venn diagram shows the number of proteins differentially expressed between the MDA-MB-231 control (Ctrl) and p300 KD cells and between the P19 WT and p300 edited (EDITED) cells treated with retinoic acid (ATRA). (**B**) Gene ontology analysis for the overlapping proteins between P19 and 231 cells. (**C**) The upregulated glycolytic proteins in the MDA-MB-231 p300 KD and P19 EDITED cells are shown in a heatmap. (**D**) Western blot validation for the glycolytic proteins in P19 WT and EDITED cells. (**E**) Western blot validation for the glycolytic proteins in MDA-MB-231 Control (Ctrl) and p300 KD cells. Protein densities were quantified by ImageJ. Relative protein levels were calculated by normalizing to H3 and then by normalizing to WT or Ctrl. (**F**) qRT-PCR validation for glycolytic genes in the P19 WT and EDITED cells with ATRA treatment. * *p* < 0.05, ** *p* < 0.01, *** *p* < 0.001. The uncropped western blot figures were presented in Figure S4.

To begin our investigations on the differential roles of CBP and p300 in cellular metabolism, we performed proteomic analysis in two cell systems that we constructed, i.e., control versus p300 knockdown MDA-MB-231 (KD) cells (Figure S1B) and WT versus p300 edited (EDITED) P19 cells [30], treated with all-trans retinoic acid (ATRA). The P19 EDITED cells were generated by deletion of 9aa in exon 2 of p300, which plays a critical role in controlling crosstalk between the nuclear receptor family and the Wnt signaling cascade and the initiation of a feed-forward differentiation mechanism [30]. Of the 976 and 2007 differentially regulated proteins in the KD and EDITED cells, respectively, there were 450 overlapping differentially regulated proteins common to the two groups (Figure 1A). Interestingly, GO analysis of the overlapping proteins demonstrated that two of the top five were metabolic pathways, specifically, glycolysis and gluconeogenesis (Figure 1B). Despite the fact that MDA-MB-231 cells are triple negative breast cancer cells and even the wild type cells are highly glycolytic, proteins critical to glycolysis, including ALDOA, ENO1 and G3P, were all significantly upregulated in the p300 KD cells (Figure 1C). To further validate our proteomic analysis, we confirmed by immunoblotting, in both the KD and EDITED cells treated with ATRA, increased expression of these proteins critical for glycolysis (Figure 1D,E). P19 EDITED cells treated with ATRA also displayed significantly enhanced expression of the key glycolytic enzymes *Aldoa*, *Aldoc*, *Eno1*, *Eno2* and *Pgam1* compared to the wild type ATRA treated P19 cells, as judged by qPCR (Figure 1F. These results imply that reduction in p300 N-terminal functionality, either by decreasing the levels of p300 by knockdown or by editing critical highly evolutionarily conserved amino terminal residues that are divergent between CBP and p300, enhances the expression of key glycolytic enzymes. 

### 3.2. Dichotomous Roles of CBP and p300 in Mitochondrial Function, Cellular Energetics and Metabolism

**Figure 2:**
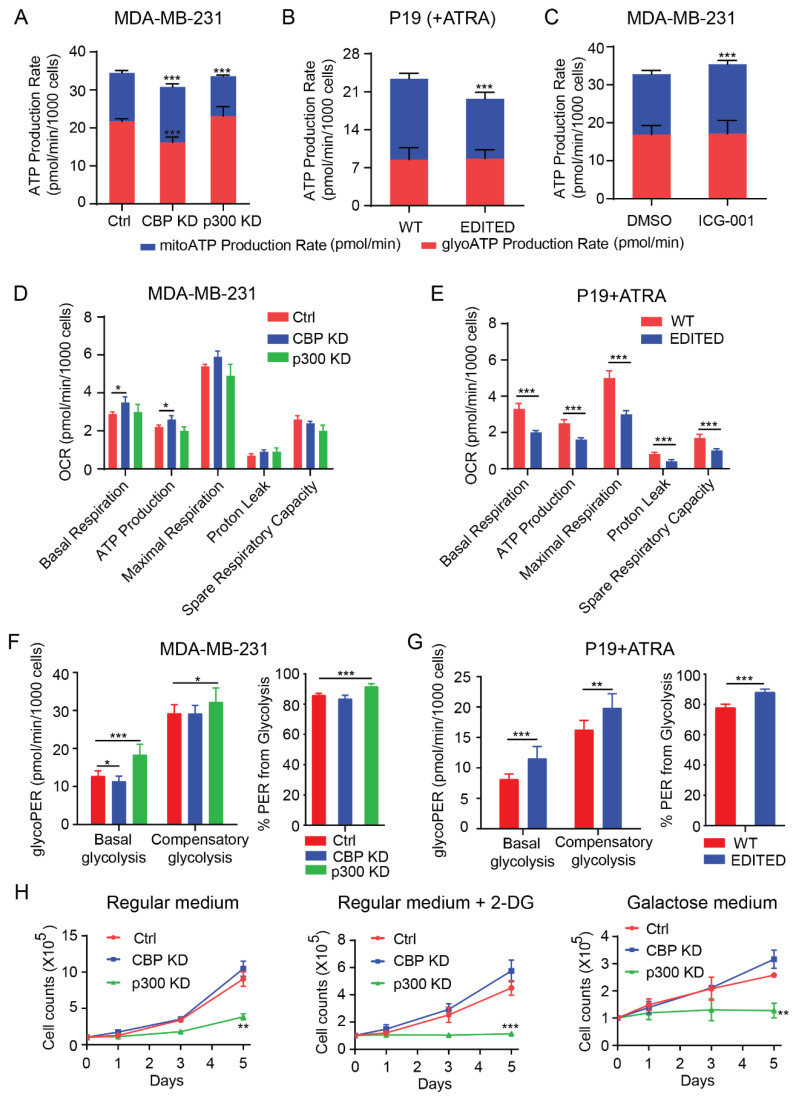
Dichotomous role of CBP and p300 in cellular metabolism and energetics. (**A**–**C**) Bar graph shows the proportion of ATP generated from mitochondrial OXPHOS or glycolysis, as determined by Seahorse ATP rate assay, in MDA-MB-231 control (Ctrl), CBP KD and p300 KD cells (**A**); in P19 WT and EDITED cells treated with ATRA (**B**); and in MDA-MB-231 control (Ctrl) and 10 µM ICG-001 treated cells (**C**). All significance testing was performed compared to control. (**D**) Bar graphs display details about mitochondrial respiratory capacities for MDA-MB-231 control (Ctrl), CBP KD and p300 KD cells. (**E**) Bar graphs show details regarding mitochondrial respiratory capacities for P19 WT and EDITED cells treated with ATRA. Glycolytic capacities are shown in bar graphs for MDA-MB-231 control (Ctrl), CBP KD and p300 KD cells (**F**), and P19 WT and EDITED cells treated with ATRA (**G**). (**H**) Cell growth rate assays for MDA-MB-231 control (Ctrl), CBP KD and p300 KD cells cultured in different media, including regular DMEM medium, DMEM with 2-DG and galactose medium. All significance tests were performed compared to control. * *p* < 0.05, ** *p* < 0.01, *** *p* < 0.001.

Proteomic analysis demonstrated that differentially expressed proteins upon knockdown or editing of p300 are important in cell metabolism. Therefore, we next sought to explore, via metabolic profiling, the functional effects of differential Kat3 coactivator usage on cell metabolism utilizing Seahorse analysis. ATP rate assay in MDA-MB-231 cells demonstrated that p300 knockdown cells generated an even greater percentage of their ATP from glycolysis, compared to the wild type control cells (Figure 2A), whereas ATP generation in CBP knockdown cells (Figure S1B) favors mitochondrial OXPHOS compared to either the p300 knockdown or wild type MDA-MB-231 cells (Figure 2A). Similarly, P19 WT cells treated with ATRA showed a greater dependence on ATP generation from mitochondrial OXPHOS, compared to the P19 p300 edited cells (Figure 2B). The small molecule CBP/β-catenin antagonist ICG-001 specifically binds to the amino terminus of CBP, thereby increasing p300 coactivator usage by β-catenin [42]. Treating MDA-MB-231 cells with ICG-001, thereby decreasing the ability of CBP’s N-terminus to function as a transcriptional coactivator, enhanced mitochondrial OXPHOS (Figure 2C), consistent with the results observed upon global CBP knockdown. To further investigate the differential roles of CBP and p300 on mitochondrial respiration, we performed Mito stress tests in MDA-MB-231 cells. Compared with either wild type control cells or p300 knockdown cells, CBP knockdown cells displayed the highest basal respiration rate, ATP production and maximal respiration. However, p300 knockdown cells demonstrated slightly decreased levels of the above mitochondrial activities compared to the already highly glycolytic wild type triple negative breast cancer cells (Figure 2D and Figure S2A). Next, we performed Mito stress tests on P19 WT and EDITED cells treated with ATRA. As anticipated, similar results demonstrating significantly decreased mitochondrial activity, as judged by all parameters, were observed upon editing the N-terminus of p300 (Figure 2E and Figure S2B). Consistent with the above analysis, p300 KD and P19 EDITED cells demonstrated enhanced glycolysis, compared to their respective controls (Figure 2F,G and Figure S2C,D). These results highlight the contrasting roles of CBP and 300 and, in particular, the N-terminal domains of the Kat3 coactivators in regulating cellular metabolism and metabolic phenotype and that disrupting p300 functionality promotes glycolytic metabolism. Cell metabolism plays an important role in multiple cellular functions, including cell proliferation. To examine this aspect, we cultured the MDA-MB-231 wild type control, CBP KD and p300 KD cells, in either control DMEM medium, DMEM medium containing 2-deoxyglucose (2-DG), which inhibits glycolysis, or galactose medium, which encourages cellular OXPHOS [43]. All three cell lines proliferated in control DMEM, albeit the p300 KD cells less rapidly. Although both the wild type and CBP KD cells proliferated more slowly in both DMEM containing 2-DG or galactose medium compared with control DMEM, CBP knockdown cells behaved similarly to the wild type cells, whereas the p300 KD cells essentially did not proliferate under these conditions (Figure 2H). This defect in proliferation in the p300 KD cells is consistent with a defect in the metabolic plasticity of these cells to utilize mitochondrial OXPHOS to proliferate under environmental conditions that restrict the use of glycolysis for energetic demands.

### 3.3. CBP and p300 Differentially Regulate Cellular Energetics during Cell Differentiation

**Figure 3:**
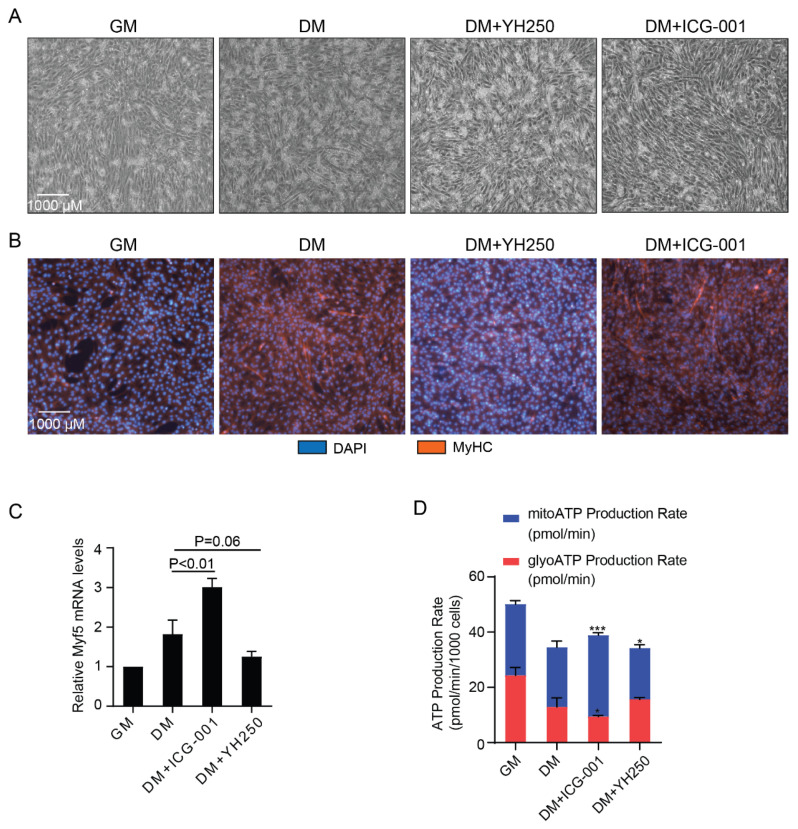
CBP and p300 differentially affect C2C12 differentiation and cellular energetics. (**A**) Cell morphology for C2C12 cells cultured in growth medium (GM), differentiation medium (DM), DM + YH250 (1 µM) and DM + ICG-001 (5 µM). Images were taken under 10× microscope. (**B**) Immunofluorescence staining for DAPI and MyHC examine the formation of C2C12 multi-nucleated myotubes and MyHC expression in growth medium (GM), differentiation medium (DM), DM + YH250 (1 µM) and DM + ICG-001 (5 µM). Images were taken under 10× fluorescence microscope. (**C**) Relative mRNA expression levels of the differentiation marker *Myf5* were examined by qRT-PCR. (**D**) Seahorse ATP rate assay for C2C12 cells cultured in growth medium (GM), differentiation medium (DM), DM + YH250 (2 µM) and DM + ICG-001 (5 µM). All significance tests were performed by comparing to DM. * *p* < 0.05, *** *p* < 0.001.

The process of cell differentiation requires energy remodeling [44,45]. We previously have shown that p300/β-catenin interactions and, in particular, phosphorylation of p300 serine 89 plays a critical regulatory role in embryonic stem cell [38] and adult progenitor cell differentiation [35]. To examine whether differential usage of CBP and p300 during cell differentiation also regulates energetic remodeling, we utilized C2C12 adult muscle satellite cells. C2C12 myoblasts differentiate into multinucleated myotubes when cultured in DMEM containing 2% horse serum (differentiation media, DM) [35]. After two days of induction, the elongated morphology of multinucleated C2C12 myotubes and the expression of the differentiation marker Myosin Heavy Chain (MyHC) was observed (Figure 3A,B). Notably, when the cells in DM were treated with the specific, small molecule direct N-terminal binding p300/β-Catenin antagonist YH250, which we have previously shown to maintain stem cell potency [46,47], differentiation as judged by myotube formation and MyHC was blocked. However, to the contrary, as anticipated [35,48], the specific CBP/β-Catenin antagonist ICG-001 [42], accelerated and enhanced differentiation (Figure 3A,B). We also examined the relative expression of the myogenic marker *Myf5*, a Wnt/β-Catenin regulated gene [49], under the same conditions described above. As judged by *Myf5* expression, cells treated with ICG-001 showed the highest level of expression, whereas the cells treated with YH250 demonstrated only a minor increase in *Myf5* expression compared to the C2C12 myoblasts maintained in growth media, consistent with the differentiation status observed by microscopy (Figure 3C). Previous reports have shown that cellular differentiation requires mitochondrial OXPHOS [50,51,52]. In that event, we utilized the Seahorse ATP rate assay during C2C12 differentiation in the presence or absence of ICG-001 or YH250. C2C12 undifferentiated proliferating myoblasts support their energetic demands relatively evenly between glycolysis and mitochondrial OXPHOS, whereas their energetic demands, as they differentiate, are more substantially met by mitochondrial OXPHOS, which is further increased by treatment with the specific CBP/β-Catenin antagonist ICG-001. Treatment with YH250, which blocks C2C12 differentiation, also decreased the reliance of ATP generation via OXPHOS (Figure 3D), compared to vehicle control or ICG-001 treated C2C12 cells in differentiation media. Taken together, our results further validate the critical importance of differential Kat3 coactivator usage in regulating and coordinating differentiation and metabolism and, in particular, that p300/β-Catenin mediated transcription is required to enhance mitochondrial OXPHOS during the initiation of cellular differentiation [4].

### 3.4. CBP and p300 Play Differential Roles in Mitochondrial Biogenesis and Mitochondrial Activity

**Figure 4:**
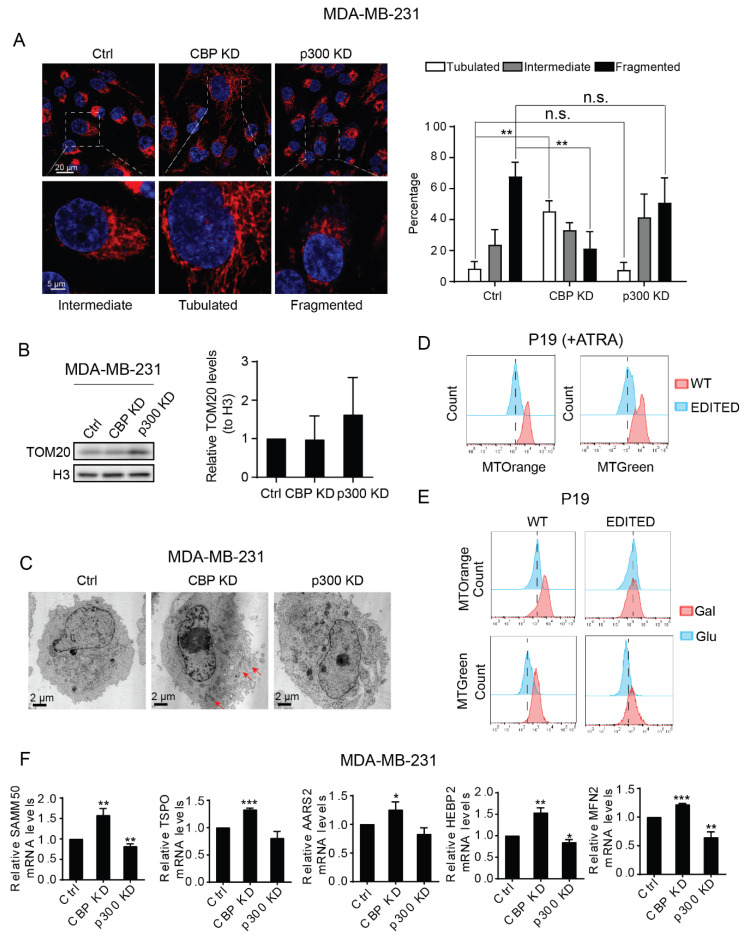
Differential roles of CBP and p300 in mitochondrial biogenesis and activity. (**A**) Immunofluorescence staining for TOM20 in MDA-MB-231 cells (left panel) and quantification of the percent of cells containing tubulated, intermediate and fragmented mitochondria (right panel). At least 100 cells were quantified for each cell line. ** *p* < 0.01, n.s. not significant. (**B**) Expression of the mitochondrial membrane protein TOM20 was examined in MDA-MB-231 control (Ctrl), CBP KD and p300 KD cells. (**C**) Transmission electron micrographs of the ECM show mitochondrial morphology in MDA-MB-231 cells. Red arrows indicate elongated mitochondria in CBP KD cells. (**D**) Mitochondrial activity and mass were investigated by FACS utilizing MitoTracker Orange and MitoTracker Green in P19 WT and P19 p300 edited (EDITED) cells treated with ATRA. (**E**) P19 WT and P19p300 edited (EDITED) cells were cultured in Glucose (Glu) or Galactose (Gal) medium. The mitochondrial activity and mass were investigated via MitoTracker Orange and MitoTracker Green by FACS. (**F**) Relative mRNA expression levels were examined for mitochondria related genes, including SAMM50, TSPO, AARS2, HEBP2 and MFN2. * *p* < 0.05, ** *p* < 0.01, *** *p* < 0.001.

Mitochondrial fusion and fission are two opposing processes that maintain the dynamic mitochondrial network [53,54]. Mitochondrial fusion and the formation of elongated mitochondria is associated with more efficient ATP production [53]. Therefore, we next investigated mitochondrial morphology in control wild type MDA-MB-231, CBP KD and p300 KD cells. Utilizing TOM20 immunofluorescence staining, our results demonstrated that the CBP KD cells displayed the highest percentage of cells exhibiting mitochondrial fusion (Figure 4A). Mitochondria in wild type MDA-MB-231 cells are already highly fragmented and we did not observe any significant change in the percentage of fragmented mitochondria in p300 KD cells by fluorescence imaging (Figure 4A). However, the p300 KD cells showed greater expression of TOM20, compared with either wild type control or CBP KD cells, consistent with more fragmented mitochondria (Figure 4B). Utilizing transmission electron microscopy, more elongated mitochondria were clearly observed in CBP KD cells compared to WT or p300 KD cells (Figure 4C). However, the total number of mitochondria among these cells was not significantly different, which was further confirmed by the relative expression of mtDNA as determined by qPCR (Figure S3A). These results indicate that by decreasing CBP, thereby promoting p300 utilization, enhanced mitochondrial fusion with a concomitant increase in mitochondrial activity. MitoTracker dyes have been previously utilized to explore multiple aspects of mitochondrial biology. MitoTracker Orange provides a readout of mitochondrial activity, whereas MitoTracker Green serves as a proxy for mitochondrial content [55]. P19 WT and P19 EDITED cells were treated with ATRA to induce differentiation. In the event, the WT P19 cells displayed more activated mitochondria, as judged by both increased MitoTracker Green and Orange intensity (Figure 4D and Figure S3B). We next tested the mitochondrial activity of P19 WT versus P19 EDITED cells cultured in either glucose or galactose medium. In the WT cells, mitochondrial activity increased in galactose medium, which requires the cells to utilize OXPHOS (Figure 4E and Figure S3C). However, the P19 EDITED cells did not show increased mitochondrial activity in galactose medium, consistent with the importance of p300 transcriptional regulation in coordinating differentiation with an energetic shift to mitochondrial OXPHOS. To further examine the differential roles of CBP/β-Catenin and p300/β-Catenin mediated transcription in mitochondrial function, by qRT-PCR, we investigated the expression of a number of mitochondrial genes, including *SAMM50*, *TSPO*, *AARS2*, *HEBP2* and *MFN2* in control wild type MDA-MB-231, CBP KD and p300 KD cells. Our results demonstrated that these genes were all slightly downregulated in the p300 KD cells and all significantly upregulated in CBP KD cells compared to control wild type cells (Figure 4F). Mitofusin 2 (MFN2) is essential for mitochondrial fusion, and its upregulation in the CBP KD cells is consistent with the above observations that knockdown of CBP promotes mitochondrial fusion. These results, in total, demonstrate that CBP knockdown promotes mitochondrial energetics, whereas p300 knockdown suppresses, further confirming their differential roles in energy metabolism.

### 3.5. P300 S89, a Critical Signaling Nexus Integrating Cellular Differentiation and Metabolism

**Figure 5:**
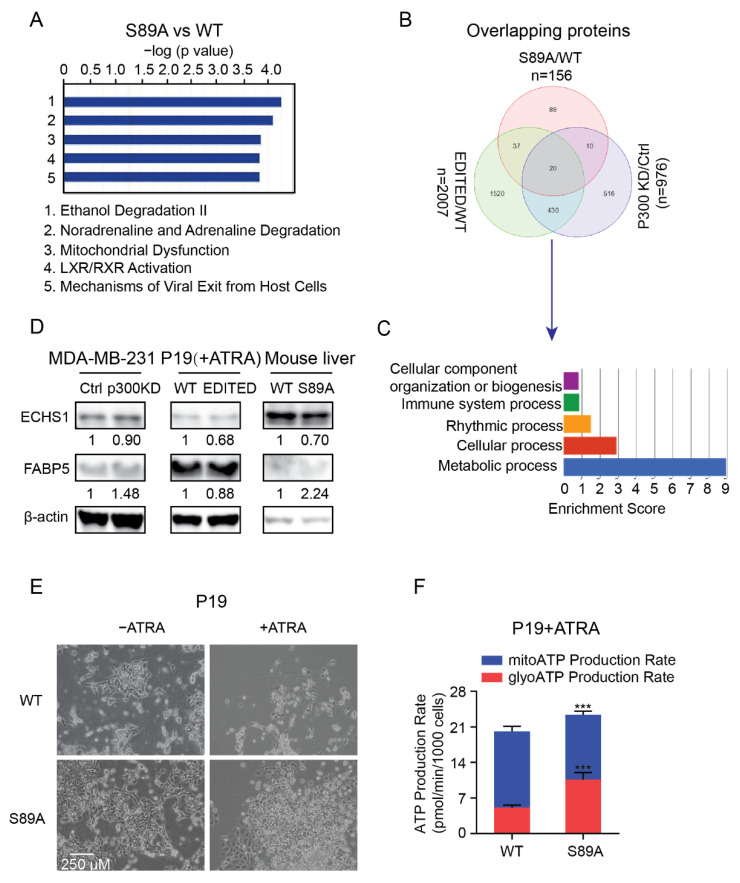
The p300 S89A point mutation affects differentiation and cellular energetics. (**A**) Gene ontology analysis for the differentially expressed proteins identified by proteomic analysis of liver tissue from the p300 S89A and WT mice. (**B**) Venn diagram depicts the differentially expressed overlapping proteins among S89A vs. WT mice, MDA-MB-231 control (Ctrl) vs. p300 KD cells and the P19 WT vs. P19 p300 edited (EDITED) cells treated with ATRA. (**C**) Gene set enrichment analysis of common overlapping 20 proteins showing the top 5 enriched GO biological process pathways. (**D**) Western blot validation for the ECHS1 and FABP5 in MDA-MB-231 control (Ctrl) and p300 KD cells, ATRA treated P19 WT and EDITED cells and liver tissues from WT and S89A C57Bl mice. Protein densities were quantified by ImageJ. Relative protein levels were calculated by normalizing to H3 and then by normalizing to WT or Ctrl. (**E**) Cell morphological images captured at 10× contrast microscope showing the differentiation of P19 WT cells, but not P19 S89A point mutated cells, after 1 µM ATRA treatment for 6 days. (**F**) Bar graph showing the proportion of ATP generated from mitochondrial OXPHOS or glycolysis, as determined by Seahorse ATP rate assay, in P19 WT and P19 S89A cells treated with 1 µM ATRA. All the significance tests were conducted by comparing to control. *** *p* < 0.001.

Our results above demonstrate that editing nine amino acids in the N-terminal region of p300 affects its ability to couple transcriptional regulation with differentiation and metabolic energetics. We have previously demonstrated the critical role that p300 serine 89 plays in adult progenitor cell differentiation [35]. To further explore this critical amino acid residue in the N-terminus of p300, we generated a S89A knockin point mutation in exon 2 of the EP300 gene in both the P19 cell line and in mice [39], via CRISPR/Cas9 gene editing and site-specific mutagenesis, respectively. This mutation removes the highly conserved phosphorylation site at the 89th amino acid of p300, which serves as an integration point for a number of signaling pathways, by changing from serine to alanine [33,34,36,39]. Gene ontology analysis of proteomic data from a number of tissues, including liver and intestine, from WT and S89A knockin mice indicated that mitochondrial dysfunction was one of the most enriched pathways (Figure 5A). Gene set enrichment analysis was performed on the 20 common differentially expressed proteins in MDA-MB-231 control vs. p300 KD cells, P19 WT vs. P19 EDITED cells and WT vs. S89A liver. Interestingly, by far the most enriched pathway was metabolic processes. Cellular processes, circadian rhythm, immune processes and biogenesis were also enriched (Figure 5B,C). To further confirm the changes observed by proteomic analysis, we examined the levels of 2 of the 20 commonly differentially expressed proteins, Enoyl-CoA Hydratase, Short Chain 1 (ECHS1) and Fatty Acid Binding Protein 5 (FABP5) in MDA-MB-231 control vs. p300 KD cells, P19 WT vs. P19 EDITED ATRA treated cells and WT vs. S89A liver by immunoblotting. ECHS1 functions in the second step of mitochondrial fatty acid beta-oxidation [56] and FABP5 is an intracellular carrier for long-chain fatty acids and can selectively deliver specific fatty acids from the cytosol to the nucleus, wherein they activate nuclear receptors [57,58]. Consistent with the proteomic analysis, differential expression of both ECHS1 and FABP5 was observed in all three systems (Figure 5D). As anticipated, based on our earlier results with the nine amino acid P19 EDITED cells [30], differentiation was dramatically inhibited in the P19 S89A mutated cells compared with the WT cells, as judged by cell morphology (Figure 5E). As previously discussed, differentiation needs to be coupled with metabolic remodeling. Consistent with this, Seahorse ATP assay indicated that the S89A mutation significantly affected the ratio of ATP generation from glycolysis and mitochondrial OXPHOS, with a significantly heavier reliance on glycolytic metabolism in the edited cells (Figure 5F). These results demonstrate that disturbing even this single amino acid in the N-terminal region of p300 dramatically interferes with cellular differentiation and transcriptional regulation of coordinated metabolic remodeling and enhanced mitochondrial OXPHOS, both in vitro and in vivo [35,38,39].

## 4. Discussion

Originally, the major roles of metabolism were considered to be the conversion of food into energy and building blocks for macromolecules and the elimination of metabolic waste. However, more recently, it has become clear that cellular metabolism is tightly connected to multiple cellular processes and the orchestration of the expression of gene cassettes that control these critical processes, including quiescence and activation of stem cells, tissue homeostasis and repair after injury, immune and inflammatory responses, tumor immunosurveillance, cell migration, development and lineage commitment and senescence [1,2,3,16]. Metabolic pathways provide the molecules necessary for gene regulation, e.g., NAD^+^, SAM-e and Acetyl-CoA [1] as well as ATP, the primary fuel driving gene expression. However, cells must constantly adjust their metabolism based upon highly variable metabolite and energy budgets dependent on cellular status, i.e., quiescent versus activated, and nutrient and oxygen availability within their microenvironment. The molecular mechanisms that connect metabolic function with the regulation of gene expression in cells to control critical cellular states, including quiescence, activation, proliferation, migration and differentiation, are just beginning to come into focus [1]. The integration of nutrient availability and metabolism and the coupling of energy production with cellular status is highly evolutionarily conserved. For example, *Caenorhabditis elegans,* when confronted with nutrient deprivation interrupt their reproductive cycle to enter the dauer state, which is regulated via the integration of carbohydrate metabolism and redox state [59], and changes in mitochondrial metabolism in pluripotent stem cells in planaria are associated with differentiation and organismal regeneration [60]. The evolution of vertebrates, with complex body plans and relatively long-lived adult lives, required a mechanism for long-term homeostatic maintenance and tissue repair. This necessitated high-fidelity regulation of somatic stem cells (SSC) to either maintain quiescence or become activated and proliferate. This was achieved via the coupling of the differential metabolic states of these two populations to protect the integrity of the genetic material in SSC [4,29]. Gene duplication, just prior to the vertebrate radiation over 450 million years ago, generated the two Kat3 coactivator family members CBP and p300 [4,19], which still maintain an extremely high degree of identity [20,21]. The extreme N-termini of the Kat3 coactivator family members, by far the least homologous regions with only 66% identity, have been the focus of our labs’ attention over the past 20 years [4]. Despite the significant divergence of the two Kat3 coactivators from one another within their N-terminal (111 amino acid) regions, 98% identity at the amino acid level has been maintained within each orthologous group within these regions for, minimally, the past 100 million years [4]. This region of the Kat3 coactivator family provides a nexus to integrate multiple signaling cascades to coordinate metabolism, inflammatory response and the regulation of cellular status via the ability to bind members of the nuclear receptor family, interferon activated Stat1 and transcriptionally competent β-catenin [4,26]. Over the years, we have extensively explored the dichotomous outcomes associated with differential Kat3 coactivator usage pharmacologically, utilizing specific CBP/β-catenin and p300/β-catenin antagonists [4,35,38,42,46,47,61,62] and, more recently, utilizing gene editing techniques both in vitro [30] and in vivo [39]. These studies, taken in total, demonstrate that differential Kat3 N-terminal coactivator usage is critically associated with the initiation of cellular differentiation (p300) or maintenance of cellular status (CBP). These pleiotropic effects are likely associated with β-catenin’s ability to recruit CBP and/or p300 to play critical roles at enhancers and super-enhancers (SE) [63,64], which can act as platforms to integrate information from multiple transcription factors [65]. The differential recruitment of CBP or p300 to SE has been observed in a number of studies, including during human myoblast differentiation [24], cellular senescence [66], immune cell function [67] and at the single-cell level in a target gene specific manner in mouse embryonic fibroblasts [68], thereby coordinately regulating transcription to couple metabolism and cellular status.

We now report utilizing multiomic and functional analyses, that differential Kat3 coactivator usage also regulates major metabolic pathway differences including lipid metabolism, oxidative stress response, mitochondrial function and oxidative phosphorylation, both in vitro and in vivo. Thus, highlighting the critical role of this evolutionarily conserved signaling nexus in coupling metabolism and energy production to cellular state and function.

We demonstrated using p300 knockdown MDA-MB-231 cells and p300 N-terminally edited P19 cells [30] that reducing p300 N-terminal functionality enhances the transcription and expression of key glycolytic enzymes (e.g., ALDOA and ENO1) (Figure 1C–F). Interestingly, obese individuals with non-alcoholic fatty liver disease express increased levels of ALDOA and ENO compared to obese controls [69] and enhanced expression of glycolysis-related genes is associated with tumor immune resistance [70]. Furthermore, the dichotomous roles of the N-termini of CBP and p300 affect mitochondrial function, cellular energetics and metabolism, as judged by Seahorse assays. Decreased N-terminal p300 functionality dramatically affects cellular metabolic plasticity, particularly under conditions disfavoring glycolysis (2-DG) or favoring OXPHOS (galactose medium) (Figure 2A–H). Interestingly, MDA-MB-231 triple negative breast tumor growth and migration, both in vitro and in vivo, are dramatically affected by knockdown of p300 [71]. The lack of tumor cell migration and invasion associated with an inability of the KD (but not wild type or scrambled knockdown cells) to effectively utilize OXPHOS, is consistent with a recent report based upon the single-cell RNA sequencing and patient-derived xenograft models of breast cancer that mitochondrial oxidative phosphorylation is the top pathway upregulated in micrometastases and that pharmacological inhibition of oxidative phosphorylation dramatically attenuated metastatic seeding [41,72,73]. 

These p300 KD clones generated in our laboratory, in a previously published report, demonstrated a dramatically reduced ability to grow when subcutaneously injected into the hind flanks of female NOD-scid IL2rγ^null^ (NSG) mice compared to either WT MDA-MB-231 cells or a scrambled control cell line. Utilizing the GFP tag from the shRNA vectors, tumor cells from both the scrambled and p300 KD xenografts were collected. The cells recovered from the xenografts were then analyzed by FACS. As opposed to the scrambled KD cells, which were primarily in G_0_/G_1,_ somewhat surprisingly the vast majority of the p300 KD cells were in G_2_/M, implying a block in mitotic progression [74,75]. However, when the p300 KD cells were put back into culture in vitro, the G2/M arrest was removed, and they demonstrated a similar cell cycle profile to the scrambled cells [71]. These results highlight the role of p300 in cell proliferation and tumor growth in vivo, particularly in a tumor microenvironment that is not particularly enriched in nutrients, where the metabolic plasticity of the tumor and the ability to utilize more energetically efficient OXPHOS is important. Crosstalk between the cell division machinery and mitochondrial dynamics and metabolism has been previously reviewed [76]. Specifically, the G2/M phase cyclin A- and cyclin B-Cdk1 complexes have been shown to phosphorylate components of mitochondrial respiratory chain complex I, thereby increasing ATP generation and promoting that the G2/M transition and impaired phosphorylation of mitochondrial complex I subunits delays cell cycle progression [77]. Wnt-dependent stabilization of proteins, referred to as Wnt/STOP, is restricted to the G2 and M phase of the cell cycle and occurs independently of β-catenin-mediated gene transcription and has been proposed to be critical to mitotic progression [78,79,80]. Wnt-induced accumulation of K48-linked polyubiquitinated proteins in endolysosomal organelles may contribute to Wnt/STOP by preventing normal degradation of Lys48-polyubiquitinated proteins in proteasomes [81]. Protein ubiquitination was the top affected pathway from GO analysis of the 450 overlapping differentially regulated proteins common to both the p300 KD MDA-MB-231 cells and P19 EDITED cells (Figure 1A). Interestingly, a very recent publication revealed that Wnt/STOP signaling is also associated with neuronal progenitor cell differentiation [82]. This is consistent with the role of p300/β-catenin signaling in cellular differentiation and neuronal differentiation in particular [48,62], via transcriptional regulation of both Wnt/STOP and non-canonical Wnt/PCP signaling [83]. Although further investigation will be required, “non-canonical” aspects of Wnt/p300/β-catenin transcription appear to be critical regulators of Wnt/STOP and mitosis. 

We have previously demonstrated the critical role that p300 and, in particular, phosphorylation of p300 serine 89 plays in the differentiation of embryonic stem cells [38,46] and adult progenitor cell, including C2C12 myoblasts [35,39]. Utilizing Seahorse metabolic analysis, we now show that undifferentiated C2C12 proliferating myoblasts support their energetic demands relatively evenly between glycolysis and mitochondrial OXPHOS. However, as they differentiate and switch Kat3/β-catenin coactivator usage to p300, as judged by increased expression of the Wnt/β-catenin regulated myogenic differentiation marker *Myf5*, their energetic demands are more substantially met by mitochondrial OXPHOS, which is further enhanced by treatment with the specific CBP/β-catenin antagonist ICG-001. Meanwhile, treatment with the direct N-terminal p300/β-catenin antagonist YH250, which blocks C2C12 differentiation, decreased ATP generation reliance on OXPHOS (Figure 3D). We further show that decreasing CBP and promoting p300 utilization enhanced mitochondrial fusion with a concomitant increase in mitochondrial activity (Figure 4). Finally, we demonstrate that disturbing even a single amino acid in the N-terminal region of p300 (i.e., serine 89 to alanine) interferes with cell differentiation and the requisite coordinated metabolic remodeling, both in vitro and in vivo. 

## 5. Conclusions

Taken together, our results highlight the critical role of the N-termini of CBP and p300 as a signaling nexus to orchestrate cellular status and metabolism and the rationale for their evolutionary conservation. We have previously proposed that a bias in CBP/β-catenin associated transcription at the expense of p300/β-catenin associated transcription with aging fits with epidemiologic data, demonstrating an increased risk of developing cancer, fibrosis, metabolic disease and neurodegeneration [4,84,85]. We have previously connected this aging associated transcriptional bias with decreased asymmetric stem cell division [4,62,86,87]. Aging is also associated with a decline in mitochondrial function and the accumulation of dysfunctional mitochondria, resulting from decreased mitochondrial oxidative phosphorylation [88], increased oxidative stress and DNA damage [89] and modulation of mitochondrial respiration mitigates metabolic syndrome of aging [40]. Therefore, rebalancing the equilibrium between CBP/β-catenin versus p300/β-catenin associated transcription may be able to ameliorate the aging process through both maintenance of our somatic stem cell pool as well as more generally via enhancing mitochondrial oxidative phosphorylation.

## Data Availability

All data described are presented in the figures and all reagents and materials are described in this section.

## Supplementary Materials

The following are available online at https://www.mdpi.com/article/10.3390/cancers13235884/s1, Figure S1: Disrupted p300 function favors glycolytic cell metabolism, Figure S2: Dichotomous role of CBP and p300 in cellular metabolism and energetics, Figure S3: Differential roles of CBP and p300 in mitochondrial biogenesis and activity, Table S1: qPCR primer sequences used in this research. Figure S4: Uncropped western blot figures.
