# Supplementary material for: Differential Kat3 Usage Orchestrates the Integration of Cellular Metabolism with Differentiation
Source: Cancers (Basel). 2021 Nov 23;13(23):5884. doi: 10.3390/cancers13235884 (PMC8656857; doi:10.3390/cancers13235884)
Supplement: Supplementary file 1 [file cancers-13-05884-s001.zip › cancers-1435597-supplementary.pdf]

Supplementary Materials: Differential Kat3 Usage Orchestrates the Integration of Cellular Metabolism with Differentiation

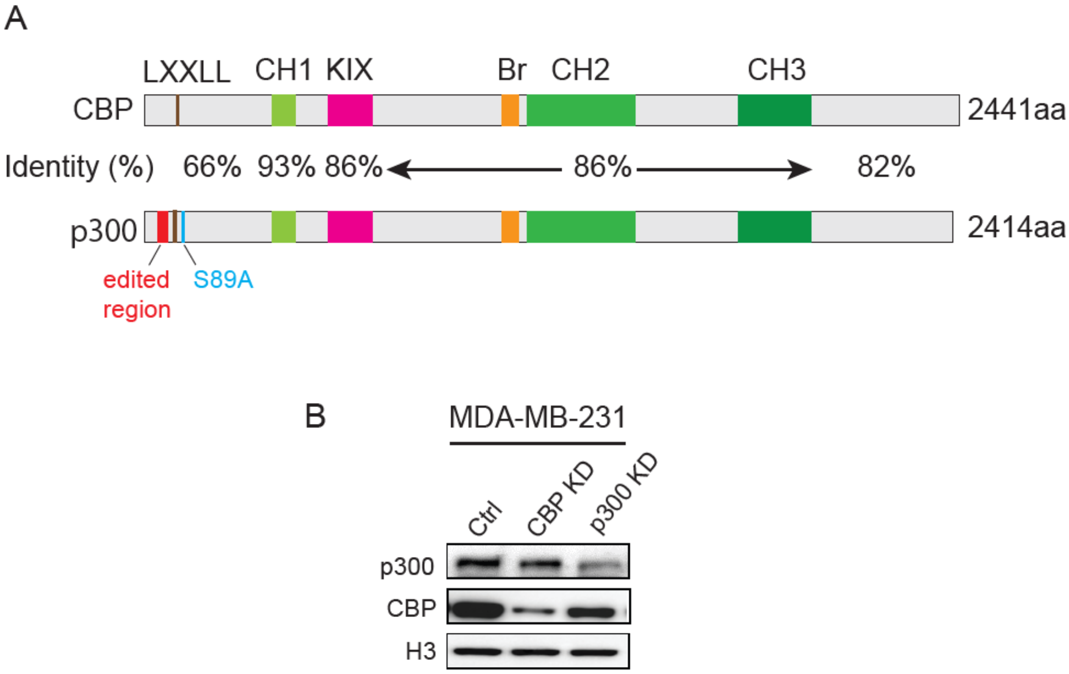

Figure S1. Disrupted P300 function favors glycolytic cell metabolism. **(A)** Schematic view of CBP and p300 protein domains showing their identity. LXXLL motif, binding site of nuclear receptor; CH, Cysteine/histidine; KIX, Kinase-inducible domain interacting domain; Br, Bromodomain; edited region indicating the region deleted in p300 protein in P19 cells. **(B)** CBP and p300 knockdown efficiency determined by western blot. The uncropped western blot figures were presented in Figure S4.

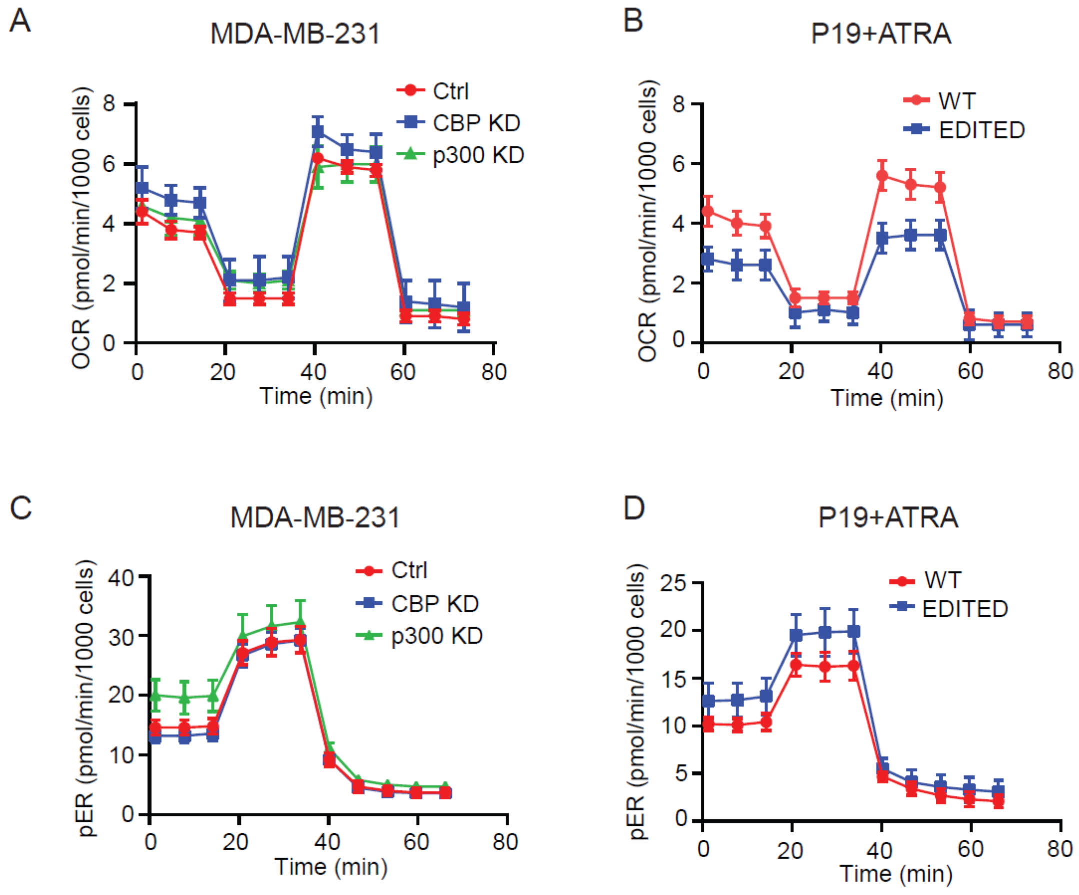

Figure S2. Dichotomous role of CBP and p300 in cellular metabolism and energetics. **(A)** Oxygen consumption rate (OCR) curves of MDA-MB-231 control (Ctrl), CBP KD and p300 KD cells by mitochondrial stress test. **(B)** OCR curves of P19 WT a nd P19 EDITED cells treated with ATRA by mitochondrial stress test. **(C)** Proton efflux rate (PER) of MDA-MB-231 control (Ctrl), CBP KD and p300 KD cells by glycolytic rate assay. **(D)** PER of P19 WT and EDITED cells treated with ATRA by glycolytic rate assay.

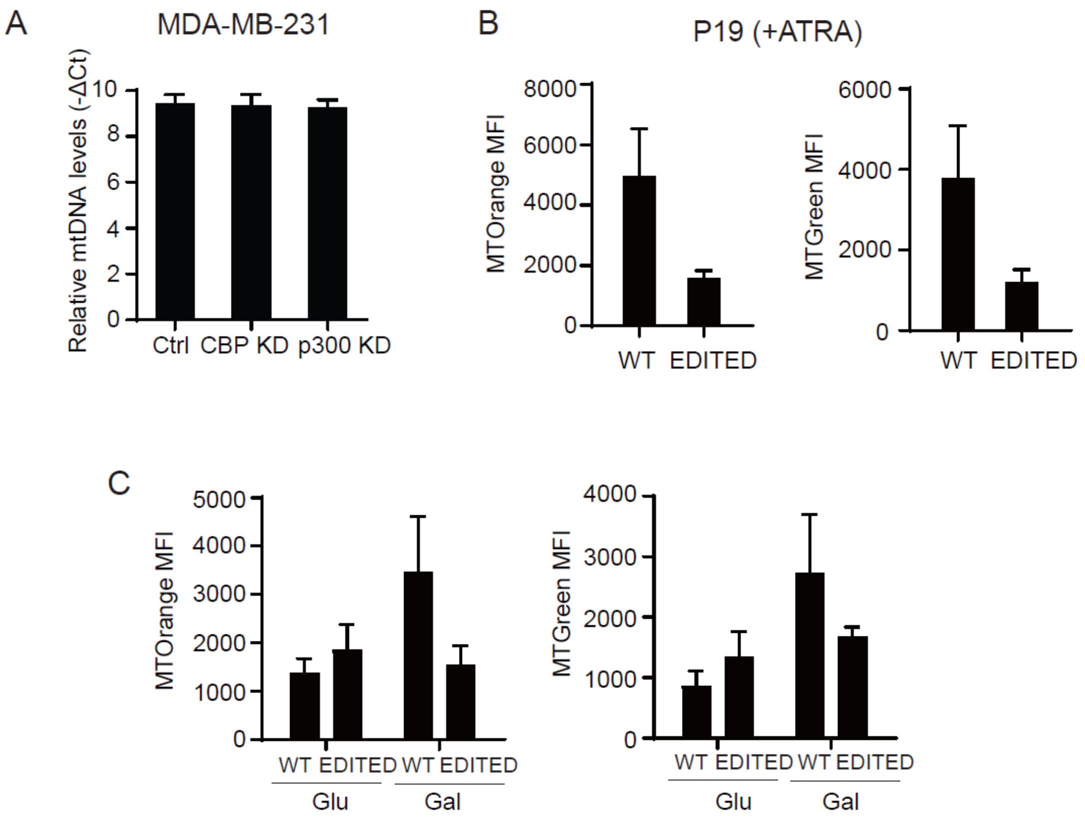

Figure S3. Differential roles of CBP and p300 in mitochondrial biogenesis and activity. **(A)** Relative mtDNA levels tested by qPCR in MDA-MB-231 control (Ctrl), CBP KD and p300 KD cells. **(B)** Bar gaphs show the MFI for MitoTracker Orange staining (left panel) and MitoTracker Green staining (right panel) in P19 WT and EDIITED cells treated with ATRA in Figure 4D. **(C)** Bar graphs show the MFI for MitoTracker Orange staining (left panel) and MitoTracker Green staining (right panel) in P19 WT and EDIITED cells cultured in glucose (Glu) or galactose (Gal) medium in Figure 4E. MFI: Median Fluorescence Intensity.

Table S1 qPCR primer sequences

**Gene symbol**	**Forward (5'-3')**	**Reverse (5'-3')**	**Species**
Eno2	GGAGAAACGGAGGACACGTT	AAGGGGATCACAGCACACTG	Mouse
Eno1	TCACAGGCTGTTGAGCACAT	GTGCCGTCCATCTCGATCAT	Mouse
Aldoa	TAGTCCTTTCGCCTACCCACC	CTCTGTCTGTTGCTGGGTGTT	Mouse
Aldoc	GGAAAAGTGAGCTGTGCTGTG	GCTGCCTACGGACTCATCTG	Mouse
Pgam1	CATGGTGAGGCCCAGGTAAA	CATTCCAGAAGGGCAGTGCT	Mouse
SAMM50	AATTCTTGAAAACAAAGATGTGGTTG	AGATTTCCGCATTACCTCAATTAGG	Human
TSPO	TCTACTCAGCCATGGGGTACG	GTCGGGCACCAAAGAAGATGG	Human
AARS2	TGGGGGTGAATATTTTAAGGAGGAG	GGGTCAAAGTAGGAGATC	Human
HEBP2	CACTATGGACCAGCCAAGTG	GTCATCTTTATTTTCATCTCTTTCTCG	Human
MFN2	CCACCTTCCTTGAAGACACGT	CTCACTGATGCCTCTCACTTTG	Human
Myf5	CCACCTCCAACTGCTCTGAC	CTTTTATCTGCAGCACATGCATTTG	Mouse
GUSB	CGTCCCACCTAGAATCTGCT	TTGCTCACAAAGGTCACAGG	Human
Gusb	TATGGAGCAGACGCAATCCC	TTCGTCATGAAGTCGGCGAA	Mouse

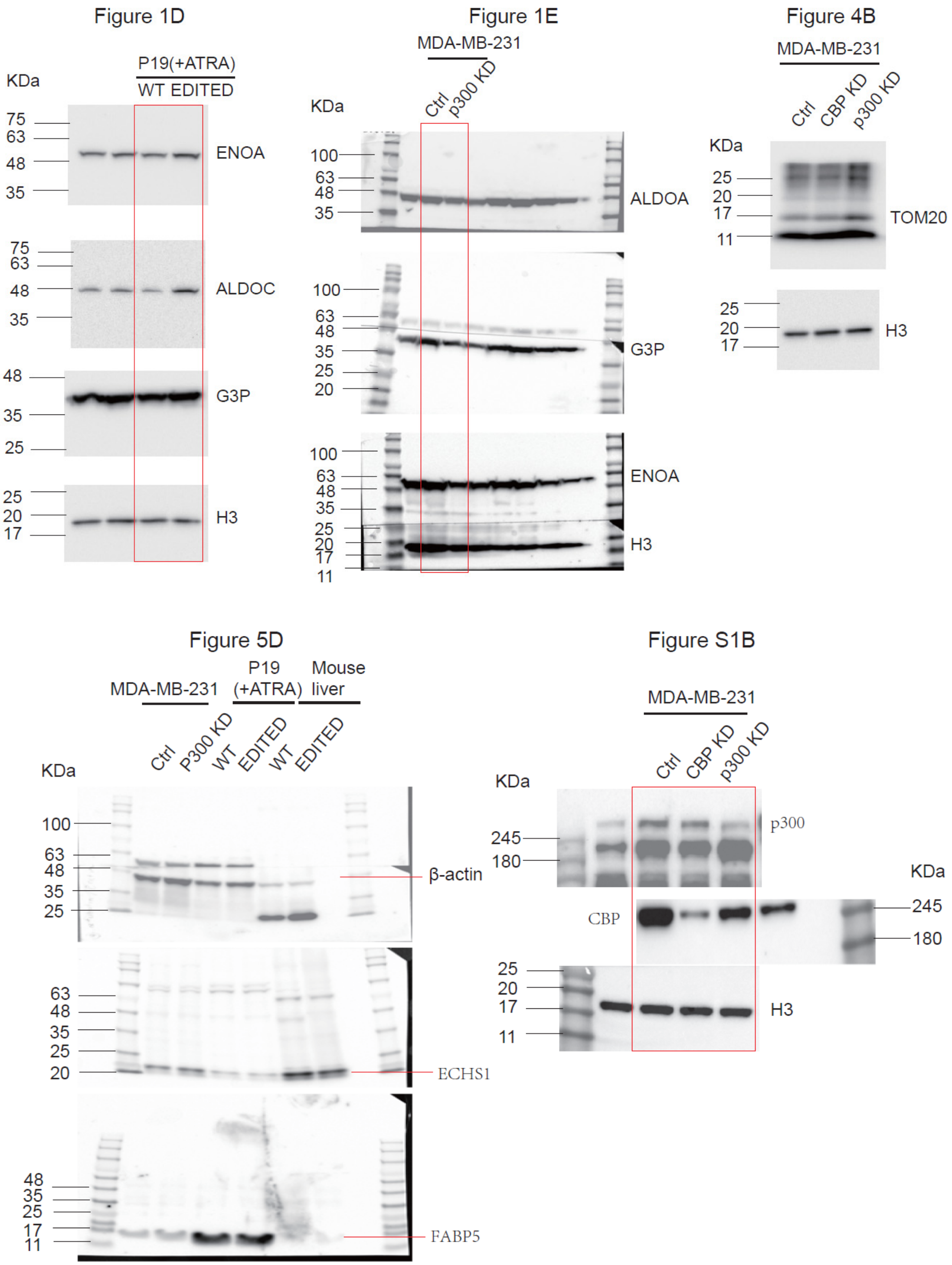

Figure S4. Uncropped western blot figures.
